# Melatonin Protects Cholangiocytes from Oxidative Stress-Induced Proapoptotic and Proinflammatory Stimuli via miR-132 and miR-34

**DOI:** 10.3390/ijms21249667

**Published:** 2020-12-18

**Authors:** Ewa Ostrycharz, Urszula Wasik, Agnieszka Kempinska-Podhorodecka, Jesus M. Banales, Piotr Milkiewicz, Malgorzata Milkiewicz

**Affiliations:** 1 Department of Medical Biology, Pomeranian Medical University, 71-111 Szczecin, Poland; e.ostrycharz@wp.pl (E.O.); wasikula@gmail.com (U.W.); milkiewm@pum.edu.pl (M.M.); 2 Department of Liver and Gastrointestinal Diseases, Biodonostia Health Research Institute-Donostia University Hospital-Ikerbasque, CIBERehd, University of the Basque Country (UPV/EHU), 20014 San Sebastian, Spain; jmbanales@unav.es; 3 Translational Medicine Group, Pomeranian Medical University, 71-210 Szczecin, Poland; p.milkiewicz@wp.pl; 4 Liver and Internal Medicine Unit, Medical University of Warsaw, 02-097 Warsaw, Poland

**Keywords:** melatonin, primary biliary cholangitis, micro RNA, oxidative stress, apoptosis

## Abstract

Biosynthesis of melatonin by cholangiocytes is essential for maintaining the function of biliary epithelium. However, this cytoprotective mechanism appears to be impaired in primary biliary cholangitis (PBC). MiR-132 has emerged as a mediator of inflammation in chronic liver diseases. The effect of melatonin on oxidative stress and bile acid-induced apoptosis was also examined in cholangiocyes overexpressing miR506, as a PBC-like cellular model. In PBC patients the serum levels of melatonin were found increased in comparison to healthy controls. Whereas, in cholangiocytes within cirrhotic PBC livers the melatonin biosynthetic pathway was substantially suppressed even though the expressions of melatonin rate-limiting enzyme aralkylamine N-acetyltransferase (AANAT), and CK-19 (marker of cholangiocytes) were enhanced. In cholangiocytes exposed to mitochondrial oxidative stress melatonin decreased the expression of proapoptotic stimuli (PTEN, Bax, miR-34), which was accompanied by the inhibition of a pivotal mediator of inflammatory response Nf-κB-p65 and the activation of antiapoptotic signaling (miR-132, Bcl2). Similarly, melatonin reduced bile acid-induced proapoptotic caspase 3 and Bim levels. In summary, the insufficient hepatic expression of melatonin in PBC patients may predispose cholangiocytes to oxidative stress-related damage. Melatonin, via epigenetic modulation, was able to suppress NF-κB signaling activation and protect against biliary cells apoptotic signaling.

## 1. Introduction

Primary biliary cholangitis (PBC) is a chronic cholestatic condition characterized by the presence of antimitochondrial autoantibodies (AMAs) and the accumulation of antigen-specific autoreactive B and T cells followed by the secretion of cytokines and biliary epithelial cell destruction [1]. The abnormal immune response during the development of PBC leads to the activation and expansion of autoreactive T and B lymphocytes followed by the production of numerous inflammatory mediators [2,3]. The ongoing inflammation in PBC patients is accompanied by a cascade of destructive events including oxidative stress followed by cholangiocyte apoptosis [4,5]. The cytoprotective mechanism against free radicals appears to be impaired in PBC. This is partially due to the decreased expression of nuclear factor-erythroid 2-related factor 2 (Nrf-2), a transcription factor that triggers an antioxidative response with the concurrent overexpression of miR-34 and miR-132, which are known to directly target the Nrf-2 gene [6].

MiR-34 and miR-132 belong to small, highly conserved, short, non-coding RNAs (microRNAs) and have pleiotropic effects on cellular homeostasis. Injured hepatocytes release reactive oxygen species (ROS) and inflammatory mediators, and miR-132 has emerged as a mediator of inflammation in chronic liver diseases. The upregulation of miR-132 has been described for the whole liver as well as in Kupffer cells isolated from mice following chronic ethanol administration [7] or bile duct ligation [8]. Similarly, miR-34 expression has been found to increase in fibrotic liver diseases in both mice and humans [6,7,8,9]. Moreover, miR-34 and miR-132 are involved in the regulation of apoptosis. MiR-132 protects against apoptosis via the downregulation of phosphatase and tensin homolog (PTEN), a well-recognized inductor of apoptosis [10,11]. In contrast, miR-34 is known to trigger apoptosis via the translational repression of either SIRT1, a deacetylase that inhibits several proapoptotic proteins including p53, or the antiapoptotic protein B-cell lymphoma-2 (Bcl-2) [12,13].

Melatonin (N-acetyl-5-methoxytryptamine) is a hormone primarily produced by the pineal gland, and its endocrine actions include regulating circadian rhythms and maintaining tissue homeostasis. Besides central nervous system melatonin is also produced in various cell types and peripheral tissues, including biliary epithelium, where it exerts paracrine and autocrine effects on cells [14,15,16]. Within cells, melatonin is present in different subcellular compartments, with especially high levels in the nucleus, cell membrane, and mitochondria where free radicals are generated [17]. High concentrations of melatonin also are present in bile, as it is essential for maintaining the function of the biliary epithelium via the activation of the antioxidant response and modulation of apoptosis [18]. The biosynthesis of melatonin is tightly regulated by aralkylamine N-acetyltransferase (AANAT), which is expressed in cholangiocytes and, to a lesser extent, in hepatocytes. Melatonin is known to modulate the innate immune response via the modulation of cytokine production [19], and it inhibits TNF-alpha and IL-6 expression by decreasing the expression of nuclear factor-kappa B (NF-κB) subunits, p50, and p65 [20]. Furthermore, melatonin protects against oxidative-stress damage either directly by scavenging free radicals and decreasing NO production, or indirectly, via the activation of Nrf-2 and SIRT1 signaling pathways [21,22]. Additionally, accumulated evidence suggests that melatonin is an apoptosis modulator; however, this effect depends on cellular context [23,24,25].

The objective of this study was to explore the expression of melatonin and its rate-limiting enzyme AANAT in the serum and livers of PBC patients. Additionally, the therapeutic effects of melatonin on oxidative stress-induced apoptosis was analyzed in vitro in human cholangiocyte (H69 cells), as well as in cholangiocytes overexpressing miR-506 (H69 miR-506), which induce PBC-like features such as an enhanced free radical generation, predisposition to bile-salt-driven apoptosis, and alterations in mitochondrial functioning.

## 2. Materials and Methods

### 2.1. Serum Samples and Liver-Tissue Specimens

This study was performed in the Liver and Internal Medicine Unit, Medical University of Warsaw between 2016 and 2018. It includes both in- and outpatients diagnosed with PBC according to the European Association for the Study of the Liver [26] criteria. Serum samples from patients with PBC (*n* = 84) and healthy individuals (*n* = 58) were collected between 8.00 and 9.00 am and stored at −80°C. Non-cirrhotic liver specimens (*n* = 22) were obtained through percutaneous needle liver biopsy (and immersed in RNAlater solution) from PBC patients who underwent liver biopsies for histological assessment (early-stage PBC). Samples of cirrhotic liver tissue were collected from PBC patients (*n* = 24) with histologically diagnosed cirrhosis (cirrhotic PBC) who underwent liver transplantation at Queen Elizabeth Hospital in Birmingham (historical samples from 2000 to 2001) and Hospital of Medical University of Warsaw (between 2014 and 2015). Control liver tissues (*n* = 22) with no microscopic changes indicative of liver disease as identified by a pathologist, were secured from large, margin liver resections of colorectal metastases specimens, as already described [6,27,28]. Demographic and clinical data on analyzed patients are summarized in Table 1 Written, informed consent was obtained from each patient included in the study. The study protocol was approved by the Ethics Committee of the Pomeranian Medical University (BN-001/43/06) and adhered to the ethical guidelines of the 1975 Declaration of Helsinki (6th revision, 2008).

### 2.2. Serum Samples and Liver-Tissue Specimens Quantitative Analyses of Melatonin Protein Expression

The concentration of melatonin was estimated is sera (Cloud-Clone Corp. #CEA908G), and liver tissue (Cusabio # CSB-E08132h) according to the manufacturers’ protocols. 

### 2.3. Cell Culture

H69 (non-tumor, SV40-immortalized, human cholangiocytes), H69-miR-506 (H69 cells with experimental constitutive overexpression of miR-506) [29], and normal human cholangiocytes (NHC) [30] were grown in media containing DMEM/F12 supplemented with fetal bovine serum, penicillin/streptomycin, vitamin solution, MEM solution, CD lipid concentrate, L-glutamine, soybean trypsin inhibitor, insulin/transferrin/selenium-A, bovine pituitary extract, epidermal growth factor, 3, 3′5-triiodo-L-thyronine, dexamethasone, and forskolin. The medium for H69-miR-506 was additionally supplemented with blasticidin (Invitrogen) for miR-506 positive selection52. To induce oxidative stress, cells were incubated with either 30 µM tert-Butylhydroquinone (tBHQ) or 250 µM H_2_O_2_. The effect of melatonin (1 mM) on microRNA levels were assessed after 16 h of incubation. Apoptosis was induced using 100 µM sodium glycochenodeoxycholate (GCDCA), and the protective effect of melatonin (500 µM) was evaluated based on the expression of apoptotic markers, i.e.; cleaved caspase 3 (CC3) and Bim. After 24 h of incubation, proteins were isolated from the cells and the levels of CC3 and Bim was assessed via immunoblot. Experiments were repeated four times.

### 2.4. MicroRNA and mRNA Extraction and Quantification

Total RNA was isolated from patient livers using the RNeasy Mini kit (Qiagen, Germantown, MD, USA), according to the manufacturer’s protocol. cDNA synthesis was carried out using the SuperscriptTM II RT kit (Invitrogen, Thermo Fisher Scientific) according to the manufacturer’s protocol. The transcripts of AANAT, TNF-α, PTEN, Bax, Bcl-2, p65, Nrf-2, and human 18sRNA were measured using Gene Expression Assays and the 7500 Fast Real-Time PCR System (Applied Biosystems, Thermo Fisher Scientific). Briefly, each assay comprised a 20 μL reaction mixture that contained 10 μL TaqMan® Gene Expression PCR Master Mix (Applied Biosystems), 2 μL diluted cDNA template, and 1 μL of the probe/primer assay mix.

For microRNA quantification, total RNA was isolated using the miRNeasy Mini Kit (Qiagen), and cDNA was synthesized using the TaqMan *Advanced miRNA cDNA Synthesis Kit* (Applied Biosystems) according to the manufacturer’s protocol. The levels of miR-34 and miR-132 (along with miR-191-5p, which was used as a endogenous control) were measured using TaqMan® Advanced miRNA Assays (Applied Biosystems). Fluorescence data were analyzed using 7500 Software v2.0.2. (Applied Biosystems), and the expression of microRNA and target genes were calculated using the ΔΔCt method of relative quantification.

### 2.5. Protein Expression Analysis

Proteins from cirrhotic PBC liver tissues and NHC cells were extracted via homogenization in an ice-cold RIPA buffer (50 mM Tris-HCl pH = 8, 150 mM NaCl, 1% NP-40, 0.5% NaDOC, 0.1% SDS, 1 mM EDTA, 100 mM PMSF, and 100 mM NaF), which contained a protease inhibitor cocktail and PhosSTOP (Roche Diagnostics GmbH). Proteins were quantified using a bicinchoninic acid assay (Micro BCA™ Protein Assay Kit; Thermo Scientific). Protein extracts (100 μg) from each liver sample were electrophoresed on SDS polyacrylamide gels and subsequently blotted onto PVDF membranes (Thermo Scientific) under semi-dry transfer conditions. After blocking for 1 h at room temperature in TBST containing 5% (*w*/*v*) milk (Merck, Gernsheim, Germany), the membranes were probed with the following primary antibodies: anti-AANAT (1:500 dilution; Santa Cruz Biotechnology, Santa Cruz, CA, USA), anti-TNFα (1:1000 dilution; Santa Cruz Biotechnology), or anti-glyceraldehyde-3-phosphate dehydrogenase (GAPDH) (1:5000 dilution; Santa Cruz Biotechnology), anti-CC3 (1:500 dilution; Cell Signaling Technology, Danvers, MA, USA), and anti-Bim (1:1000 dilution; Cell Signaling Technology). For the detection of antigen-antibody complexes, a peroxidase-conjugated anti-rabbit secondary antibody (1:5000 dilution; AmershamTM, GE Healthcare, Waukesha, WI, USA) or an anti-mouse secondary antibody (1:50,000 dilution; Jackson ImmunoResearch, West Grove, PA, USA) was used. Protein expression was detected using an enhanced chemiluminescence detection system (Chemiluminescent HRP Substrate, Millipore). Bands were visualized and quantified using the MicroChemi 2.0 System and GelQuant software (DNR Bio-Imaging Systems Ltd., Neve Yamin, Israel).

### 2.6. Immunohistochemistry

Frozen liver sections (6 µm) derived from patients with PBC and controls were fixed in a methanol and acetone mixture (1:1) at −20 °C for 5 min. The cut order of sections was known and described. We examined the proteins of interest through immunohistochemistry analyses. Briefly, sections were treated with Avidin/Biotin Blocking Kit (#SP-2001; Vector Laboratories, USA) and then incubated in 3% H202 diluted in methanol. Sections were probed with rabbit anti-AANAT (1:500 dilution; Santa Cruz Biotechnology), anti-TNFα (1:1000 dilution; Santa Cruz Biotechnology), and anti-CK19 (1:50 dilution; Santa Cruz Biotechnology). Next, sections were incubated with either biotinylated anti-mouse/anti-rabbit IgG (Vector Laboratories) or biotinylated anti-goat IgG (Vector Laboratories). Reactions were visualized using ABC Vectastain and DAB kits (DAKO, Agilent Technologies, Denmark). The negative controls, for which the primary antibodies were omitted, were included in the analysis and uniformly demonstrated no reaction.

### 2.7. Statistics

Data were evaluated as mean ± standard error (SE) for continuous variables and further analyzed using (one-way/two-way) ANOVAs. A *p*-value < 0.05 was considered statistically significant. Stat-View-5 Software (SAS Institute, USA) was used for all analyses. Demographic and laboratory features of analyzed subjects in Table 1 are presented as mean ± standard deviation (SD).

## 3. Results

### 3.1. Melatonin Concentration is inCreased in the Serum of Patients with PBC, But Reduced in the Liver

**Figure 1:**
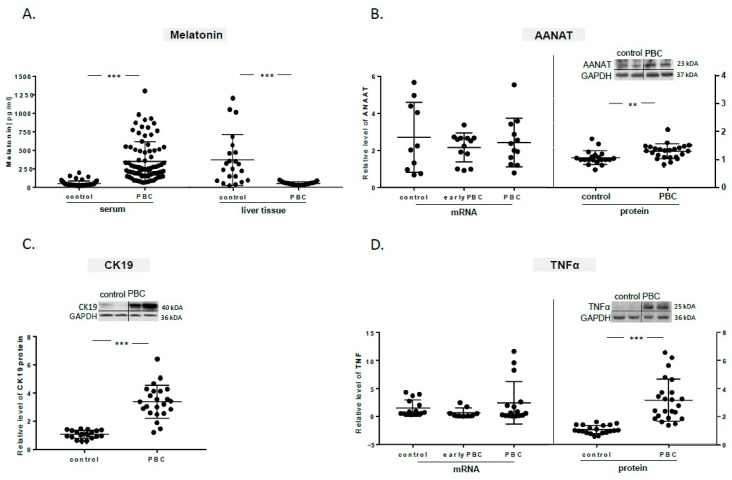
Levels of melatonin, aralkylamine N-acetyltransferase (AANAT), CK19, and TNFα in the livers of patients with primary biliary cholangitis (PBC). (**A**) Serum concentration of melatonin was increased in PBC patients, in contrast to liver tissue where the concentration of this hormone was decreased in comparison to controls. (**B**) The level of AANAT protein was enhanced, whereas the expression of mRNA was not change in either early-stage PBC or cirrhotic PBC. (**C**) The level of the CK19 protein was increased in the livers of PBC patients in comparison to controls. (**D**) The TNFα protein level was upregulated in PBC, while no changes in mRNA expression were observed in early-stage PBC or cirrhotic PBC. Melatonin concentration was evaluated using an ELISA assay, while AANAT, CK19, and TNFα mRNA and protein levels were evaluated using real-time PCR and Western blot, respectively. Values are shown as mean ± SE. ** *p* < 0.01 and *** *p* < 0.001.

The serum concentration of melatonin was 7.6-fold higher in patients with PBC than in controls (*p* < 0.001); however, the expression of this hormone in the livers of PBC patients was significantly reduced (90% reduction, *p* < 0.001 vs. controls, Figure 1A). In this regard, the mRNA expression levels of the melatonin rate-limiting enzyme AANAT were not altered between cirrhotic livers from PBC patients compared to non-cirrhotic PBC or healthy controls, whereas its protein levels were found significantly increased in the liver of patients with PBC vs. healthy controls (1.4-fold; *p* = 0.002 vs. controls; Figure 1B). On the other hand, the protein levels of CK19, a marker of cholangiocytes and hepatocytes transformed into cholangiocytes in advanced fibrosis, was elevated in the liver tissue of patients with PBC when compared to healthy controls (3.5-fold, *p* < 0.001 vs. controls; Figure 1C), indicating increased ductular reaction. Similarly, the levels of the TNFα protein was substantially increased in cirrhotic PBC livers (3-fold, *p* < 0.001 vs. controls; Figure 1D).

**Figure 2:**
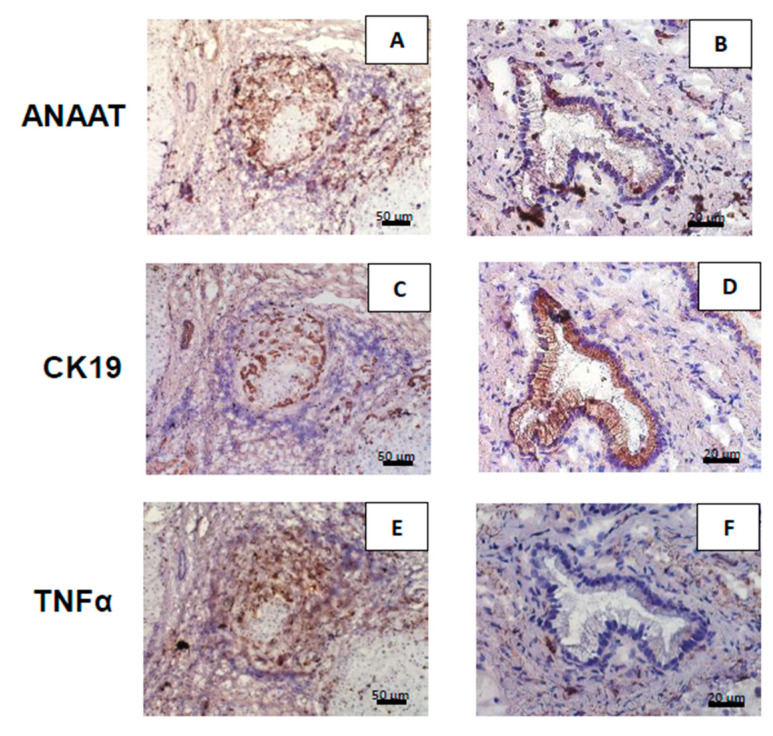
Hepatic expressions of aralkylamine N-acetyltransferase (AANAT), CK19, and TNFα protein in primary biliary cholangitis (PBC) patients. Positive immunohistochemical staining (dark brown) of AANAT, CK19, and TNFα in cirrhotic liver tissues were observed on the edges of regenerative nodules (**A**,**C**,**E**), and in bile ducts (**B**,**D**,**F**), respectively. (**A**,**B**) AANAT was ubiquitous in the liver tissue of PBC patients. (**C**,**D**) CK19 was localized in cholangiocytes in areas of ductular reactions. Bile ducts are marked by asterisks. (**E**,**F**) TNFα was localized primarily on the perimeter of nodules, but was absent in the cholangiocytes of the large bile duct.

The presence of AANAT was confirmed in the bile ducts (Figure 2A), as well as in regenerative nodules (Figure 2B). TNFα-positive cells were on the perimeter of the nodules (Figure 2E), but was barely detected in the bile ducts (Figure 2F). Antibodies against the cholangiocyte marker cytokeratin 19 (CK19) allowed for the visualization of biliary epithelial cells within bile ducts (Figure 2C) and hepatocytes transformed into cholangiocytes (Figure 2D) in cirrhotic livers.

### 3.2. Melatonin Modified the Expression of miR-132 and miR-34 in Cholangiocytes Subjected to Oxidative Stress

**Figure 3:**
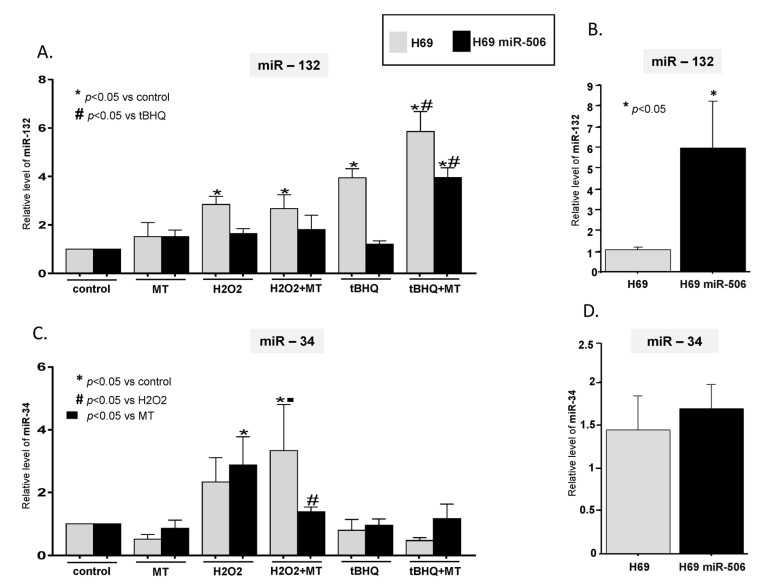
The effect of melatonin on miR-132 and miR-34 expression in cholangiocytes under conditions of oxidative stress. (**A**) Oxidative stress induced either by H_2_O_2_ or tert-Butylhydroquinone (tBHQ) enhanced the expression of miR-132 in H69 cells, whereas in H69miR-506 cells this response was only observed when the cells were treated simultaneously with tBHQ and melatonin. (**B**) The basal level of miR-132 expression was significantly increased in H69miR-506 cells in comparison to H69 cells. (**C**) H_2_O_2_ induced miR-34 expression in both H69 and H69miR-506 cells. Melatonin suppressed this enhancement, but only in H69miR-506 cells. (**D**) The baseline expression of miR-34 was comparable in H69miR-506 and H69 cells. Each experiment was repeated four times with similar results. MicroRNA were analyzed using real-time PCR, and the results were normalized to miR-191. Values are shown as mean ± SE.

The expression of miR-132 in H69 human cholangiocytes in vitro was enhanced in response to the oxidative stress induced by both H_2_O_2_ (2.8-fold vs. controls; *p* = 0.01) and tBHQ (3.9-fold vs. controls, *p* = 0.0004). Yet, in H69-miR-506 cholangiocytes this response was not observed. Melatonin had a significant effect on miR-132 expression in H69 and H69 miR-506 cholangiocytes, but only when the oxidative stress was induced by tBHQ. Thus, in H69 cells cotreatment with melatonin and tBHQ further enhanced the miR-132 level (1.5-fold vs. tBHQ, *p* = 0.01, and 5.8-fold vs. controls, *p* < 0.0001). The event was different for H69-miR-506 cells, where the oxidative stress induced by tBHQ did not alter the miR-132 level. However, when the cells were coincubated with tBHQ and melatonin, the miR-132 expression was strongly upregulated in comparison to both controls and tBHQ-treated cells (3.9-fold, *p* < 0.0001, and 3.3-fold, *p* < 0.001, respectively, Figure 3A).

The miR-34 expression was only affected by H_2_O_2_-induced cellular oxidative stress. In H69 cells, melatonin further enhanced the H_2_O_2_-induced expression of miR-34 (3.3-fold vs. controls, *p* = 0.02; 6.5-fold vs. H_2_O_2_, *p* = 0.007; Figure 3B). Incubation of H69-miR-506 cells with H_2_O_2_ resulted in the induction of miR-34, which was significantly suppressed by melatonin (50% reduction, *p* = 0.04 vs. H_2_O_2_; Figure 3C). An analysis of the baseline expression of the microRNAs showed that in H69 cells with an experimental overexpression of miR506 the expression of miR-132 was 6-fold higher than in H69 cholangiocytes (*p* = 0.04; Figure 3B). However, there was no significant difference between those two cell lines when miR-34 expression was analyzed (Figure 3D).

### 3.3. In H69 miR-506 Cells Subjected to Oxidative Stress, Melatonin Modulated Apoptosis and the Expression of Transcription Factors Regulating Inflammation

**Figure 4:**
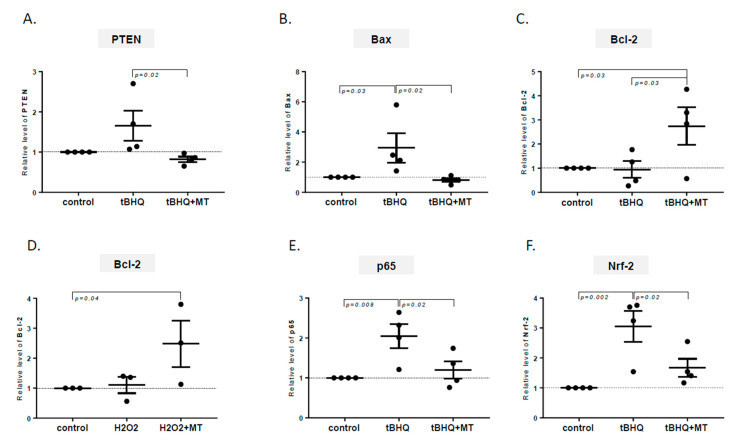
The effect of melatonin on apoptosis- and inflammation-related factors in H69-miR-506 cells subjected to oxidative stress. Melatonin inhibited the tert-Butylhydroquinone (tBHQ)-induced upregulation of proapoptotic factors (**A**) phosphatase and tensin homolog and (**B**) Bax, and induced expression of antiapoptotic factor B-cell lymphoma-2 in cells treated with (**C**) tBHQ and (**D**) H_2_O_2_. In cells subjected to mitochondrial oxidative stress, melatonin suppressed the expression of (**E**) p65, a subunit of nuclear factor-kappa B associated with inflammation, and (**F**) nuclear factor-erythroid 2-related factor 2, a marker of oxidative stress. Each experiment was repeated four times with similar results. All factors were estimated using real-time PCR. The results were normalized to 18S RNA. Values are shown as mean ± SE.

In looking for the mechanism related to the protective role of melatonin against oxidative stress in cholangiocytes, we analyzed the interplay between melatonin and the expression of anti- and proapoptotic genes. Melatonin suppressed PTEN expression in tBHQ-treated H69-miR-506 cells (*p* = 0.02 vs. tBHQ; Figure 4A). Moreover, coincubation with melatonin and tBHQ prevented the induction of proapoptotic Bax expression (3.6-fold reduction vs. tBHQ-treated cells, *p* = 0.002, and a 20% reduction vs. controls *p* = 0.03; Figure 4B) in these cells. In contrast, melatonin had the opposite effect on the antiapoptotic protein Bcl-2 and upregulated the Bcl-2 expression in cells exposed to oxidative stress induced by tBHQ (2.9-fold increase vs. tBHQ, *p* = 0.03, and 2.7-fold increase vs. controls, *p* = 0.03, Figure 4C). Furthermore, melatonin affected the level of the Bcl-2 protein when oxidative stress was induced by H_2_O_2_ (2.5-fold induction vs. controls, *p* = 0.04, Figure 4D). We also observed that incubation with tBHQ enhanced the expression of p65, the subunit of NF-κB nuclear factor (2-fold vs. nontreated cells, *p* = 0.008), and Nrf-2, the marker of oxidative stress (3-fold vs. nontreated cells, *p* = 0.002), and that this upregulation was restrained when the cells were additionally treated with melatonin (80% reduction vs. tBHQ, *p* = 0.02; and 60% reduction vs. tBHQ, *p* = 0.02; respectively, Figure 4E,F).

### 3.4. Melatonin Restrained Bile Salt-Induced Apoptosis of Cholangiocytes

**Figure 5:**
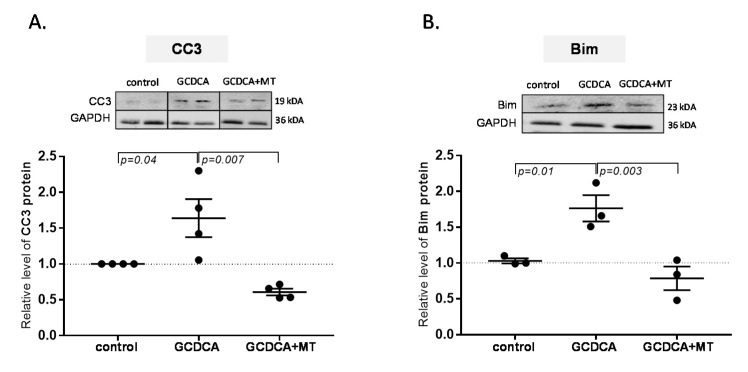
Melatonin protected against toxic bile acid-induced apoptosis in normal human cholangiocytes (NHCs). Melatonin suppressed the expression of sodium glycochenodeoxycholate (GCDCA)-induced (**A**) active form of caspase-3, cleaved caspase-3 (CC3), and (**B**) proapoptotic protein Bim. Protein levels were evaluated using Western blot and quantified relative to glyceraldehyde-3-phosphate dehydrogenase. Values are shown as mean ± SE.

Melatonin suppressed the protein level of CC3 in NHC cells incubated with GCDCA. GCDCA induced CC3 expression (1.6-fold, *p* = 0.04 vs. control; Figure 5A), which was inhibited by melatonin (2.7-fold reduction vs. GCDCA, *p* = 0.007). Similarly, the enhanced expression of the proapoptotic Bim protein was induced by GCDCA (1.76-fold, *p* = 0.01 vs. control), and melatonin cotreatment downregulated the Bim level in those cells (2.2-fold reduction vs. GCDCA, *p* = 0.003; Figure 5B).

## 4. Discussion

While the beneficial effects of melatonin using models of liver damage have been previously described, there has not, thus far, been experimental studies investigating the effect of melatonin within the context of cholangiopathy characterized by cholestasis, ductopenia, and biliary inflammation in primary biliary cholangitis.

The elevated serum concentration of melatonin in PBC patients suggests that the overproduction of this hormone in pineal sites is a compensatory mechanism to alleviate an exacerbated immune response and oxidative stress during the development of cholangitis. In contrast, the hepatic concentration of melatonin is substantially reduced in cirrhotic PBC. This implies that neither pineal-gland secreted melatonin is capable of providing sufficient protection of liver cells, nor is melatonin biosynthesis by cholangiocytes impaired. Consequently, melatonin does not elicit its endocrine and paracrine effects on cells, which are constantly subjected to inflammatory insults and free-radical generation. Moreover, in this study we showed that a low hepatic concentration of melatonin is not the result of an insufficient expression of the rate-limiting enzyme AANAT, as the protein level of this enzyme was significantly enhanced in PBC livers in comparison to controls. Furthermore, the presence of AANAT in pathologically altered liver cells, including cholangiocytes, was confirmed by immunohistochemical analysis. The function of AANAT is spatially and temporally regulated, and its activity strongly depends on post-translational modifications, notably phosphorylation [31,32]. The balance between phosphorylation and dephosphorylation of AANAT is maintained by cAMP and calcium ions, and both molecules are important in cholangiocyte biology. For instance, in PBC, cAMP in large cholangiocytes and calcium in small cholangiocytes are involved in cholangiocyte proliferation [33]. The homeostasis of both molecules may be altered due to the overexpression of miR-506, which has been reported in PBC [34]. Furthermore, miR-506 targets the type III inositol 1, 4, 5-trisphosphate receptor, and alterations of its level leads to the deterioration of calcium homeostasis [35]. Further studies are needed to investigate whether changes in the intracellular conditions in PBC cholangiocytes result in inappropriate post-translational modifications of AANAT and the inadequate biosynthesis of melatonin.

Melatonin has been demonstrated to ameliorate liver damage by decreasing oxidative stress, inflammatory responses, and biliary senescence [36,37]. In human cholangiopathies such as PBC and PSC, an initial balance between cholangiocyte apoptosis and compensatory cholangiocyte proliferation is followed by a failure in the proliferative capacity of cholangiocytes, and enhanced apoptosis favors evolution toward ductopenia. The downregulation of melatonin in the livers of PBC patients may significantly decrease the ability of cholangiocytes to counteract disease-related insults. Cholangiocytes that undergo chronic injury are more prone to apoptosis. We have previously shown that PBC is characterized by the enhanced expression of two oxidative-stress related microRNAs, namely miR-132 and miR-34, which are implicated in the modulation of apoptosis [6]. MicroRNA-132 is engaged in maintaining cellular and tissue homeostasis via the regulation of apoptosis, autophagy, and cell proliferation [11,38]. In H69 cells, the expression of miR-132 was induced following treatment with both H_2_O_2_ and tBHQ, and melatonin further enhanced its expression under tBHQ-induced oxidative stress. In contrast, in H69 miR-506 cells, neither H_2_O_2_ nor tBHQ induced oxidative stress was able to elevate the level of miR132. This may be due to the fact that the baseline expression of miR-132 was significantly higher in H69 miR-506 cells in comparison to H69 cells. It is worth mentioning that H69 miR-506 cells (a cellular model of PBC) [35] are characterized by chronic stress due to a stable overexpression of miR-506.

Intriguingly, melatonin-dependent, enhanced expression of miR-132 was observed only when cells were subjected to tBHQ, but not H_2_O_2_ incubation. The action of tBHQ is related to the mitochondria, an important site of a free-radical generation, and a key cellular compartment involved in apoptosis [39]. Melatonin acts as a free radical scavenger, and may directly inhibit the stress-mediated release of cytochrome c and, thus, block caspase activation and apoptosis by the activation of the MT1 receptor located in the outer mitochondrial membrane [40]. We undertook additional studies to demonstrate a causal link between melatonin and the development of apoptosis in response to oxidative stress. Melatonin substantially enhanced the expression of miR-132 in cholangiocytes exposed to tBHQ, which was accompanied by the downregulation of both PTEN and the proapoptotic Bax protein, along with the upregulation of the antiapoptotic protein Bcl-2. These observations indicate there is a role for melatonin in protecting against apoptosis induced by mitochondrial oxidative stress. Our study is in accordance with observations suggesting a positive effect of melatonin treatment by which the upregulation of miR-132 inhibited the PTEN-dependent proapoptotic pathway in primary cortical neurons [38]. Similarly, following ischemic neuronal injury, melatonin reverses the proapoptotic phenotype by blocking Bax activity and inducing the Bcl-2 protein [41]. The induction of miR-132-5p has been reported to upregulate the antiapoptotic protein Bcl-2 [42]. We also found that melatonin suppressed miR-34 in H_2_O_2_-treated cholangiocytes and was associated with the upregulation of antiapoptotic Bcl2. This beneficial effect of melatonin in suppressing miR-34 is important as this microRNA promotes apoptosis and senescence, inhibits proliferation, and leads to marked alterations in Bcl‑2 and p53 expressions [43,44].

Our research also provides data showing the effects of melatonin treatment in protecting against GCDCA-induced apoptosis in human cholangiocytes. High levels of BA, including GCDCA at pathophysiological levels, have been shown to injure mitochondria by changing their membrane potential and releasing cytochrome c [45,46]. Here, we showed that melatonin, via reduced levels of cleaved caspase-3 and the proapoptotic protein Bim, may protect against the development of apoptosis.

The anti-inflammatory properties of melatonin have been extensively studied in animal and cellular models [20,47]; however, its role in cholestatic liver disease needs to be further elucidated. Experimental and clinical data have suggested that melatonin reduces chronic and acute inflammation by modulating serum inflammatory factors and through the inhibition of prostanoids, leukotrienes, and nitric oxide production along with the inhibition of nuclear factor-kappa B [48,49,50]. Our data demonstrate that melatonin suppressed NF-κB signaling activation by reducing p65 expression in tBHQ-oxidative stress induced in cholangiocytes with PBC-like phenotypes. Furthermore, in this cellular model of PBC, the marker of oxidative stress, Nrf-2, was downregulated following cotreatment with melatonin. Consistent with our observations are reports showing that the inhibition of NF-κB signaling contributes to melatonin alleviating inflammation, downregulating mtROS production [51], and preventing invasiveness in HepG2 liver cancer cells [52].

In conclusion, the results of this study suggest that decreased melatonin secretion in the livers of PBC patients is not due to an insufficient hepatic expression of the AANAT enzyme and interestingly, the biosynthesis of this enzyme was even increased. The inadequate expression of melatonin predisposes liver cells to immune- and oxidative stress-related damage. Our findings demonstrate the beneficial effects of melatonin supplementation in cholagiocytes with PBC-like phenotypes. Melatonin, via epigenetic modulation, was able to suppress NF-κB signaling activation and protect against apoptotic signaling induced by either oxidative stress or high concentrations of bile salt. In view of significantly higher serum level of melatonin in patients with PBC as compared to controls, simple oral supplementation of melatonin does not seem to address this problem at the tissue level. Delivery of melatonin to the liver using a carefully selected carrier such as bile acids could be a possible venue for further investigations.

## Figures and Tables

**Table 1:** Demographic and laboratory features of analyzed subjects.

Parameters	Liver			Serum	
	Controls (*n* = 22)	PBC Early Stages (*n* = 22)	PBC Cirrhotic (*n* = 24)	Controls (*n* = 58)	PBC (*n* = 84)
Gender (M/F)	4/18	0/22	3/21	3/55	4/80
Age (mean ± SD)	50 ± 4	52 ± 11	57 ± 8	50 ± 5	51 ± 9
Hb (mean ± SD, NR 12–16 g/dL)	13.5 ± 0.5	12.8 ± 0.6	10.0 ± 1.5	14.6 ± 0.9	11.6 ± 1.8
Bilirubin (mean ± SD, NR mg/dL)	0.7 ± 0.2	1.3 ± 0.8	6.9 ± 6.7	0.6 ± 0.3	2.4 ± 2.7
ALP (mean ± SD, NR 40–120 IU/L)	83 ± 18	265 ± 182	560 ± 383	78 ± 20	455 ± 284
ALT (mean ± SD, NR 5–35 IU/L)	24 ± 12	81 ± 16	78 ± 15	22 ± 13	114 ± 101
AST (mean ± SD, NR 5–35 IU/L)	22 ± 11	62 ± 56	233 ± 291	21 ± 11	99 ± 83
Albumin (mean ± SD, NR 3.8–4.2 g/dL)	4.4 ± 0.6	4.0 ± 0.4	3.6 ± 0.6	4.3 ± 0.5	3.9 ± 0.6

Abbreviations: PBC—primary biliary cholangitis; Hb—hemoglobin; ALP—alkaline phosphatase; ALT—alanine aminotransferase; AST—aspartate aminotransferase; NR—normal range.
